# Pharmacological inhibition of EZH2 as a promising differentiation therapy in embryonal RMS

**DOI:** 10.1186/1471-2407-14-139

**Published:** 2014-02-27

**Authors:** Roberta Ciarapica, Elena Carcarino, Laura Adesso, Maria De Salvo, Giorgia Bracaglia, Pier Paolo Leoncini, Alessandra Dall’Agnese, Federica Verginelli, Giuseppe M Milano, Renata Boldrini, Alessandro Inserra, Stefano Stifani, Isabella Screpanti, Victor E Marquez, Sergio Valente, Antonello Mai, Pier Lorenzo Puri, Franco Locatelli, Daniela Palacios, Rossella Rota

**Affiliations:** 1 Department of Oncohematology, Laboratory of Angiogenesis, Ospedale Pediatrico Bambino Gesù, IRCCS, Piazza S. Onofrio 4, 00165 Rome, Italy; 2 IRCCS Fondazione Santa Lucia, Rome, Italy; 3 Departments of Pathology, Ospedale Pediatrico Bambino Gesù, IRCCS, Rome, Italy; 4 Departments of Surgery, Ospedale Pediatrico Bambino Gesù, IRCCS, Rome, Italy; 5 Centre for Neuronal Survival, Montreal Neurological Institute, McGill University, Montreal, Quebec, Canada; 6 Department of Molecular Medicine, Sapienza University, Rome, Italy; 7 Chemical Biology Laboratory, Frederick National Laboratory for Cancer Research, CCR, National Cancer Institute, NIH, Frederick, Maryland, USA; 8 Istituto Pasteur, Fondazione Cenci Bolognetti, Dipartimento di Chimica e Tecnologie del Farmaco, Sapienza University, Rome, Italy; 9 Muscle Development and Regeneration Program, Sanford-Burnham Medical Research Institute, La Jolla, California, USA; 10 Dipartimento di Scienze Pediatriche, Università di Pavia, Pavia, Italy

**Keywords:** EZH2, Histone methyltransferase, rhabdomyosarcoma, Polycomb proteins, Differentiation, DZnep, EZH2 catalytic inhibitors

## Abstract

**Background:**

Embryonal Rhabdomyosarcoma (RMS) is a pediatric soft-tissue sarcoma derived from myogenic precursors that is characterized by a good prognosis in patients with localized disease. Conversely, metastatic tumors often relapse, leading to a dismal outcome. The histone methyltransferase EZH2 epigenetically suppresses skeletal muscle differentiation by repressing the transcription of myogenic genes. Moreover, de-regulated EZH2 expression has been extensively implied in human cancers. We have previously shown that EZH2 is aberrantly over-expressed in RMS primary tumors and cell lines. Moreover, it has been recently reported that EZH2 silencing in RD cells, a recurrence-derived embryonal RMS cell line, favors myofiber-like structures formation in a pro-differentiation context. Here we evaluate whether similar effects can be obtained also in the presence of growth factor-supplemented medium (GM), that mimics a pro-proliferative microenvironment, and by pharmacological targeting of EZH2 in RD cells and in RD tumor xenografts.

**Methods:**

Embryonal RMS RD cells were cultured in GM and silenced for EZH2 or treated with either the S-adenosylhomocysteine hydrolase inhibitor 3-deazaneplanocin A (DZNep) that induces EZH2 degradation, or with a new class of catalytic EZH2 inhibitors, MC1948 and MC1945, which block the catalytic activity of EZH2. RD cell proliferation and myogenic differentiation were evaluated both *in vitro* and *in vivo*.

**Results:**

Here we show that EZH2 protein was abnormally expressed in 19 out of 19 (100%) embryonal RMS primary tumors and cell lines compared to their normal counterparts. Genetic down-regulation of EZH2 by silencing in GM condition reduced RD cell proliferation up-regulating p21^Cip1^. It also resulted in myogenic-like differentiation testified by the up-regulation of myogenic markers *Myogenin*, *MCK* and *MHC*. These effects were reverted by enforced over-expression of a murine Ezh2, highlighting an EZH2-specific effect. Pharmacological inhibition of EZH2 using either DZNep or MC inhibitors phenocopied the genetic knockdown of EZH2 preventing cell proliferation and restoring myogenic differentiation both *in vitro* and *in vivo*.

**Conclusions:**

These results provide evidence that EZH2 function can be counteracted by pharmacological inhibition in embryonal RMS blocking proliferation even in a pro-proliferative context. They also suggest that this approach could be exploited as a differentiation therapy in adjuvant therapeutic intervention for embryonal RMS.

## Background

Pediatric rhabdomyosarcoma (RMS) is a locally invasive soft-tissue sarcoma with a predisposition to metastasize that accounts for ~ 30% of all soft-tissue sarcomas (STS) and for 7-8% of all solid tumors in childhood [1]. Embryonal RMS is the major histopathologic subtype, accounting for 60% of all RMS cases and, when nonmetastatic, shows a 5-year overall survival of 70% [2]. Childhood cancer statistics show that the outcome for young patients with RMS has tremendously improved from 53% in 1975–1978 to 68% in 1979–1982 [3], but unfortunately current treatments for embryonal RMS in the metastatic form often do not respond to therapy. Indeed, metastatic or relapsed forms, even if they can undergo complete remission with secondary therapy, are often characterized by poor long-term prognosis and dismal outcome [4-6]. Moreover, children who relapse need to be closely monitored for a long time as anti-cancer therapy side effects may persist or develop months or years after treatment. Therefore, novel more specific and less toxic treatment approaches, such as molecular targeted therapies, are under study. Since RMS cells share characteristics of skeletal muscle precursors, the most reliable theory about the origin of RMS suggests that perturbations of the normal mesenchymal development of the skeletal muscle lineage might have a causative role [7]. Consistently, results from some groups and ours recently suggest that a differentiation therapy seems to represent an alternative way to reduce the aggressiveness of cancer cells, not by exerting cytotoxicity but by restoring the differentiation fate of tumor cells [8-12]. Indeed, under specific treatments, RMS cells progress toward less proliferating myoblast-like cells that are capable to develop myotube-like structure. The methyltransferase Polycomb Group (PcG) protein Enhancer of zeste homolog 2 (EZH2), the catalytic factor of the Polycomb Repressor Complex 2 (PRC2), represses gene transcription by silencing target genes through methylation of histone H3 on lysine 27 (H3K27me3) and it has been shown to prevent cell differentiation and promote cell proliferation in several tissues [13]. Increasing evidence demonstrates that EZH2 is not only aberrantly expressed in several types of human cancers, but often behaves as a molecular biomarker of poor prognosis [14-21]. EZH2 was clearly shown to act as a negative regulator of skeletal muscle differentiation favoring the proliferation of myogenic precursors [22-24]. This function results from an EZH2-dependent direct repression of genes related to myogenic differentiation [22]. We previously reported that EZH2 is markedly expressed in the RMS context, both in cell lines and primary tumors compared to their normal counterparts [25]. The first evidence of the role of EZH2 as a main player in the inability of RMS cells to undergo differentiation has been recently reported *in vitro* for the embryonal RMS cell line RD, established from a tumor recurrence, through EZH2 genetic silencing upon serum withdrawal [26].

Here, after having shown that EZH2 was de-regulated in a cohort of primary embryonal RMS, we evaluated whether it was possible to boost the differentiation capability of embryonal RMS RD cells after EZH2 inhibition even in serum-enriched culture conditions. As an additional promising approach, we investigated whether pharmacological inhibition of EZH2 in RD cells by either reducing its expression or catalytically inhibiting its activity might be detrimental for cancer cell proliferation both *in vitro* and *in vivo*. Our data demonstrate that EZH2 down-regulation restores the myogenic differentiation of RD cells with no need to reduce serum (cultured in growth medium), and that pharmacological inhibition of EZH2 is a feasible way to restrain the tumor-promoting potential in embryonal RMS.

## Methods

Additional file 1: Supplementary Methods.

### Cell lines

RD embryonal RMS cell line was obtained from American Type Culture Collection (Rockville, MD). A204 and RH18 embryonal RMS cell lines were obtained from Deutsche Sammlung von Mikroorganismen und Zellkulturen GmbH (Braunschweig, Germany). Normal Human Skeletal Muscle cells (SkMC; myoblasts) were obtained from PromoCell (Heidelberg Germany).

### Nuclear fraction-enrichment

Cells were lysed and assayed as previously reported [10]. Briefly, cells were lysed in cytoplasm lysis buffer A (10 mM HEPES pH 7.9, 10 mM KCl, 0.2 mM EDTA, 1 mM DTT), containing protease inhibitors, 0.5 mM phenylmethylsulfonylfluoride (PMSF) and 0.6% Nonidet P-40 (Sigma Chemical Co., St Louis, MO, USA). Lysates were centrifuged at 10.000 rpm 10 min at 4°C and the supernatants (cytoplasmic fractions) were split into aliquots and rapidly frozen. The nuclear pellet was washed in buffer A without Nonidet P-40 and finally resuspended in nuclear lysis buffer B (20 mM HEPES pH 7.9, 0.4 M NaCl, 2 mM EDTA, 1 mM DTT), containing protease inhibitors and 1 mM PMSF (Sigma Chemical Co., St Louis, MO, USA). Samples were incubated on ice 30 min and centrifuged at 13.000 rpm 10 min at 4°C; the supernatants (nuclear fractions) were split into aliquots and rapidly frozen or used for western blot analysis.

### Western blotting

Western blotting was performed on whole-cell lysates and histone extracts as previously described [27,28]. Briefly, cells were lysed in RIPA buffer (50 mM Tris–HCl pH7.4, 150 mM NaCl, 1 mM EDTA, 1% D.O.C. (Na), 0,1% SDS, 1% Triton X-100) containing protease inhibitors (Sigma Chemical Co., St Louis, MO, USA). Lysates were sonicated, incubated on ice 30 min and centrifugated at 10,000 g 20 min at 4°C. Supernatants were used as total lysates. Protein concentrations were estimated with the BCA protein assay (Pierce, Rockford, IL). EZH2 was detected using the EZH2 antibody (612666; Transduction LaboratoriesTM, BD, Franklin Lakes, NJ). Antibodies against Myogenin (F5D) and Myosin Heavy Chain (Meromyosin, MF20) were obtained from the Developmental Studies Hybridoma Bank at the University of Iowa (DSHB, Iowa City, IA). Antibodies against p21Cip1 (sc-397), β-actin (sc-1616) and all secondary antibodies were obtained from Santa Cruz Biotechnology (Santa Cruz Biotechnology, Inc., Santa Cruz, CA). Antibodies against Troponin I (4002) were obtained from Cell Signaling (Beverly, MA). The antibody against the Topoisomerase IIβ was obtained from Sigma Aldrich (Sigma Chemical Co., St Louis, MO, USA). Antibody against against Histone 3 (H3), H3K27me3 (Lys27) and H3K4me3 (Lys4) were obtained from Millipore (EMD Millipore Corporation, Billerica, MA, USA). Antibody against α-tubulin (ab4074) was from Abcam (Cambridge, UK). All the antibodies were used in accordance with the manufacturer’s instructions.

### Histone extraction

Cells were harvested and washed twice with ice-cold Phosphate Buffered saline (PBS) 1X supplemented with 5 mM Sodium Butyrate and resuspended in Triton Extraction Buffer (TEB: PBS, 0.5% Triton X 100 (v/v)) containing 2 mM PMSF and 0.02% (w/v) NaN3 (107 cells/ml) and lysated on ice for 10 min. Lysates were centrifuged at 2000 rpm for 10 min at 4°C and the pellets were washed in half volume of TEB and centrifuged.Histones were extracted O/N at 4°C from pellets resuspended in 0.2 N HCl (4×107 cells/ml). Samples were then centrifuged and supernatants were used for western blot analysis.

### Transient RNA interference

Cells were sequentially transfected by 2 subsequent rounds (24 h), to secure efficient cell silencing, with ON-TARGETplus SMART pool siRNA targeting different regions of the EZH2 transcript (L-004218-00) or non-targeting siRNA (control; D-001206-13), previously validated in other publications [14,29,30] (both from Dharmacon, Thermo Fisher Scientific, Lafayette, CO).

### Real time qRT-PCR

Total RNA was extracted using TRizol (Invitrogen, Carlsbad, CA) and analyzed by real-time RT-qPCR for relative quantification of gene expression [27] using Taqman gene assays (Applied Biosystems, Life Technologies, Carlsbad, CA) for GAPDH (Hs99999905_m1), EZH2 (Hs01016789_m1), Myogenin (Hs01072232_m1), MCK (Hs00176490_m1) and p21 (Hs00355782_m1). For the relative quantification of Murine Ezh2 and MHC mRNA the SYBR-green method was used (Applied Biosystems, Life Technologies, Carlsbad, CA) with primers previously reported [31] or available on request. The values were normalized to the levels of glyceraldehyde-3-phosphate dehydrogenase (GAPDH) mRNA. An Applied Biosystems 7900HT Fast Real-Time PCR System (Applied Biosystems, Life Technologies, Carlsbad, CA) was used for measurements.

### Murine Ezh2 over-expression

Flag-tagged murine Ezh2, cloned into the pMSCV retroviral vector (Addgene, Cambridge, MA) or control empty vector, both co-expressing the Green Fluorescent Protein (GFP) as reporter gene, were kindly obtained from G. Caretti. Phoenix ampho cells were obtained from ATCC and cultured in DMEM supplemented with 10% FBS (growth medium, GM).Transient transfection of Phoenix ampho cells were performed using lipofectamine reagent (Invitrogen, Carlsbad, MA) and viral particles were collected after 48 h. Supernatant containing viral particles were used to infect RD cells O/N in the presence of 8 ug/ml of polybrene.

### Immunofluorescence for MHC detection

Immunofluorescence to visualize MHC was performed as previously described using the MF-20 antibody (Developmental Studies Hybridoma Bank at the University of Iowa, Iowa City, IA) [10]. Briefly, cells were washed 3 times in PBS, fixed 10 min in 4% PFA and permealized 5 min with 0.2% Triton X-100 in PBS. After 30 min in PBS containing 3% bovine serum albumin, slides were incubated 1 h at room temperature with the MF-20 antibody against myosin heavy chain (MHC; Developmental Studies Hybridoma Bank at the University of Iowa, Iowa City, IA). After 2 washing in PBS, cells were treated with a rhodamine-conjugated secondary antibody (Millipore, Temecula, CA). After being counterstained with DAPI, chamber slides were mounted in GelMount (Biomeda, Foster City, CA, USA). Images were acquired with an Eclipse E600 fluorescence microscope, through LUCIA software version 4.81 (Nikon, Sesto Fiorentino, Firenze, Italy).

### Cell cycle and apoptosis assays

Cells were transfected 24 h after seeding (Day 0) with siRNAs and after 24 h transfected again. Then, they were harvested and counted at the reported time points. For pharmacological treatments RD cells were treated with the S-adenosyl-L-homocysteine hydrolase inhibitor 3-Deazaneplanocin A (DZNep) and MC1945 for 24 h, 48 h, 72 h and 96 h. For cell cycle assay, cells were harvested by trypsinization at the indicated time points, washed in ice-cold PBS, fixed in 50% PBS and 50% acetone/methanol (1:4 v/v) for at least 1 h and, after removing alcoholic fixative, stained in the dark with a solution containing 50 μg/ml Propidium Iodide (PI) and 100 μg/ml RNase (Sigma) for 30 min at room temperature. For quantification of apoptosis, cells were harvested, washed twice with ice-cold PBS and stained in calcium-binding buffer with APC-conjugated Annexin V and 7-Aminoactinomycin D (7-AAD) using Annexin V apoptosis detection kit (BD Pharmingen, San Diego, CA), according to manufacturer’s recommendations. Samples were analyzed within 1 h. The stained cells were analyzed for both cell cycle and apoptosis by fluorescence-activated cell sorting using a FACSCantoII equipped with a FACSDiva 6.1 CellQuest software (Becton Dickinson Instrument, San Josè, CA).

### Chromatin immunoprecipitation (ChIP)

ChIP assay was performed as previously described (70) with minor modifications. Briefly, chromatin was cross-linked in 1% formaldehyde for 15 min at room temperature and quenched by addition of glycine at 125 mM final concentration for 5 min at room temperature before being placed on ice. Cells were washed twice with ice-cold PBS containing 1 mM PMSF and 1X protease inhibitors, resuspended in ice-cold cell lysis buffer (10 mM Tris–HCl pH 8, 10 mM NaCl, 0.2% NP-40, 1 mM PMSF and 1X protease inhibitors) and incubated on ice for 20 minutes. After centrifugation at 4000 rpm for 5 min, nuclei were resuspended in ice-cold nuclear lysis buffer (50 mM TrisHCl pH 8.1; 10 mM EDTA; 1% SDS, 1 mM PMSF and 1X protease inhibitors) and left on ice for 10 min. Chromatin was then sonicated to an average fragment size of 200–300 bp using a Bioruptor and diluted ten times with IP dilution buffer (16.7 mM Tris–HCl pH 8.1, 167 mM NaCl, 1.2 mM EDTA, 0.01%SDS, 1.1% Triton X-100, 1 mM PMSF and 1X protease inhibitors). Diluted chromatin was pre-cleared using protein G-agarose magnetic beads (Invitrogen) for 1 hour at 4°C and incubated with the corresponding antibodies O/N at 4°C. The following antibodies were used: anti-acetylated histone H3, anti-trimethyl Lysine 27 histone H3 and anti-trimethyl Lysine 4 histone H3 (EMD Millipore Corporation, Billerica, MA, USA) and anti-Ezh2 (Diagenode s.a. Liège, Belgium). Immunoprecipitated chromatin was recovered by incubation with protein G-agarose magnetic beads (Invitrogen, Carlsbad, CA) for 2 hours at 4°C. Beads were washed twice with low salt washing buffer (20 mM Tris–HCl pH8, 2 mM EDTA, 1% Triton X-100, 0.1% SDS, 150 mM NaCl), twice with high salt washing buffer (20 mM Tris–HCl pH8, 2 mM EDTA, 1% Triton X-100, 0.1% SDS, 500 mM NaCl) and twice with TE before incubating them with elution buffer (10 mM Tris–HCl pH8 1 mM EDTA, 1% SDS) for 30 minutes at 65°C. Cross-linking was then reverted O/N at 65°C and samples were treated with proteinase K for 2 hours at 42°C. The DNA was finally purified by phenol: chloroform extraction in the presence of 0.4 M LiCl and ethanol precipitated. Purified DNA was resuspended in 50 μl of water. Real-time PCR was performed on input samples and equivalent amounts of immunoprecipitated material with the SYBR Green Master Mix (Applied Biosystems, Life Technologies, Carlsbad, CA). Primer sequences are available on request.

### Xenograft experiments and immunohistochemistry

Athymic 6-week-old female BALB/c nude mice (nu + \nu+) were purchased from Charles River. Procedures involving animals and their care were conformed to institutional guidelines that comply with national and international laws and policies (EEC Council Directive 86\609, OJ L 358, 12 December 1987). RD cell suspensions in PBS (10×10^6^ cells in 100 μl) were injected subcutaneously into the posterior flanks of nude mice. When the tumors became palpable, i.e., about approximately 70–80 mm^3^, mice were intraperitoneally injected with MC1945 (2.5 mg/Kg) or control vehicle (DMSO) twice daily, 3 days per week for 3 weeks when mice were sacrificed. No visible signs of toxicity such as weight loss or behavioral change were seen with the compound dose and treatment timing used, as already reported [32,33]. Tumor volume was measured by caliper with the following formula: tumor volume (mm3) = L × S2 × π/6 wherein L is the longest and S the shorter diameter and π/6 is a constant to calculate the volume of an ellipsoid, as described [10]. Representative tumor growth data were obtained from 3 mice per treatment/group. In a parallel experiment, 3 mice per treatment/group were sacrificed 12 days after the first treatment, i.e. the exponential tumor growth phase, and xenografts removed after tumor volume measurement. Portions of the excised tumors embedded in paraffin were used for immunohistochemical analysis. Sections of 10 μm cut from xenograft blocks were stained with hematoxylin/eosin. Five μm serial sections were subjected to immunohistochemistry for the expression of EZH2 and Ki67 with methods and antibodies reported below for primary human RMS samples. The MF-20 antibody (DSHB, USA) was used to detect the expression of MHC. Counterstaining was carried out with Gill’s hematoxyline (Bio-Optica, MI, Italy). Sections were dehydrated and mounted in non-aqueous mounting medium. Images were acquired under an Eclipse E600 microscope (Nikon) through the LUCIA software, version 4.81 (Nikon) with a Nikon Digital Camera DXM1200F.

### Immunohistochemistry on RMS primary tissues

Archival, de-identified formalin-fixed, paraffin-embedded RMS and control tissues were obtained from the Department of Pathology of Ospedale Pediatrico Bambino Gesù in Roma, (Italy) after approval of the Institutional Review Boards. Clinicopathological characteristics of the cohort are reported in Table 1. Histopathological features of the tumors were reviewed for the present study by a Pathologist (R. B) blinded to the results of immunohistochemical analysis. Sections from RMS samples and 3 control muscle tissues were cut at 3–5 μM, deparaffinized in xylene and rehydrated through graded ethanol. Antigen retrieval was performed for 25 min at 98°C. After endogenous peroxidase blocking with 3% H2O2 in Tris-buffered saline (TBS) for 30 min at room temperature (RT), 3% to 5% BSA in TBS was applied for 1 hour at room temperature for non-specific background blocking. Sections were treated with Biotin Blocking System (DAKO, Carpinteria, CA) for additional blocking, according to the manufacturer’s instructions. Sections were incubated with primary antibodies for EZH2 (Transduction LaboratoriesTM, BD, Franklin Lakes, NJ), as reported [34] and Ki67 (Novocastra; Newcastle upon Tyne, UK), and then with secondary antibodies EnVision System-HRP (Power vision Plus method, Zymed, San Francisco, CA, USA) and Biotinilated link (DAKO, Carpintera, CA), respectively. Positive reactions were visualized by staining with 3-amino-9-ethylcarbazolo (AEC) and 3,3′-diamminobenzidine (DAB) (DAKO Carpintera, CA), respectively, and then sections were slightly counter-stained with Gill’s hematoxylin (Bio-Optica, Milan, Italy). Negative controls were stained in parallel by treating serial cross-sections simultaneously either with isotype non-specific IgG or omitting the primary antibody. Positive staining was defined as well-localized nuclear pattern. Levels of expression were semi-quantitatively quantified by scoring the percentage of positive nuclei stained for each specific molecule per microscopic field in at least 5 fields per section by 2 blinded observers and, in rare cases of discrepancy, by an additional third independent observer. Differences in intensity of immunoreactivity were not taken into account. Each section was scored using an Eclipse E600 microscope (Nikon, Sesto Fiorentino, Firenze, Italy) at 400× magnification. Images were acquired through LUCIA software, version 4.81 (Nikon, Sesto Fiorentino, Firenze, Italy) with a Nikon Digital Camera DXM1200F.

**Table 1:** Clinical and histopathological features of pediatric patients with embryonal rhabdomyosarcoma (RMS) (n=19)

	**Embryonal RMS n (%)**
Sex	
Male	11 (58)
Female	8 (42)
Age (years)	
< 10	14 (74)
≥ 10	5 (26)
Localisation	
Orbit-genitourinary tract-head and neck^$^	9 (47)
Cranial paramenigeal-extremity-other^$$^	10 (53)
Tumor volume	
< 5 cm	7 (37)
≥ 5 cm	12 (63)
IRS stage	
I	2 (10)
II	3 (16)
III	11 (58)
IV	3 (16)
Metastasis	
No	16 (84)
Yes	3 (16)
Recurrence	
No	12 (63)
Yes	7 (37)
Outcome	
Alive	13 (68)
DOD	6 (32)
Expression of markers	
EZH2 (positive cells/microscopic field)	40 (range 29-44)
Ki67 (positive cells/microscopic field)	20 (range 17-29)

*Abbreviations*: *DOD* dead of disease, *IRS* Intergroup Rhabdomyosarcoma Study Group staging system.

^$^Favorable and ^$$^Unfavorable tumor localization.

### Statistical analysis

The Student’s t-test was done to assess the difference between various treatments. Statistical significance was set at a two-tailed P value less than 0.05. All analyses were performed with SPSS 11.5.1 for Windows Package (© SPSS, Inc., 1989–2002 and © LEADTOOLS 1991–2000, LEAD Technologies, Inc., Chicago, IL).

## Results

### EZH2 protein is expressed in embryonal RMS primary tumors

**Figure 1:**
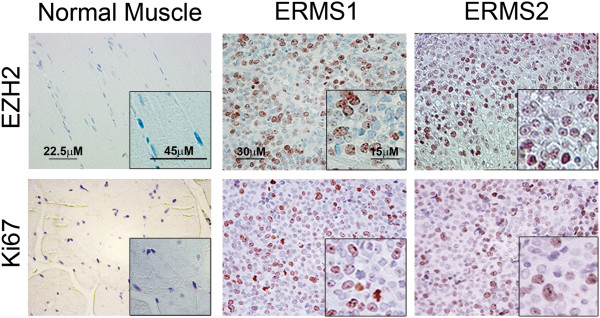
**EZH2 protein levels are up-regulated in primary embryonal rhabdomyosarcoma (RMS) tissues.** Representative immunohistochemical staining showing EZH2 (upper panels) and Ki67 (bottom panels) expression in sections of normal muscle and primary tumor tissue of two embryonal RMS specimens (RMS1 and RMS2). Brown-orange color in nuclei indicates positive staining (400× Magnification). Normal muscles are negative for both markers. Insets represent higher magnification of selected regions.

Previously, our and other groups reported that the expression of EZH2 mRNA in embryonal RMS primary tumors was markedly expressed while was not detectable in muscle tissues [25,35]. Here, we semi-quantitatively analyzed the expression of EZH2 protein by immunohistochemistry in 19 embryonal RMS primary tumors (Table 1). Strikingly, EZH2 was expressed in the nuclei of all the RMS specimens tested that are also positive for the nuclear expression of the proliferative marker Ki67 (Table 1 and Figure 1). By contrast, normal control muscles were negative for both markers (Figure 1). These findings indicate that also the expression of EZH2 protein is abnormally elevated in embryonal RMS primary tumors.

### Down-regulation of EZH2 reduces embryonal RMS cell proliferation

**Figure 2:**
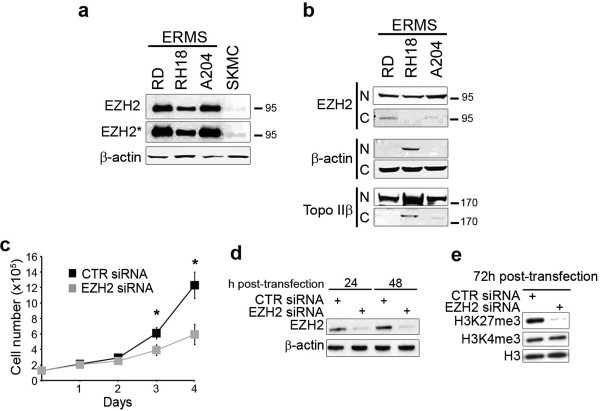
**EZH2 depletion inhibits embryonal rhabdomyosarcoma (RMS) cell proliferation. (a)** Western blot showing EZH2 and β-actin (loading control) in whole-cell lysates from embryonal RMS cell lines and normal human myoblasts SKMC as control, all cultured in proliferating growth medium (GM, i.e., supplemented with 10% fetal calf serum). EZH2* band: longer exposition. Representative of three independent experiments. **(b)** Western blot analysis of nuclear (N) and cytoplasmic (C) -enriched cell fractions of embryonal RMS cell lines. Nuclear EZH2 was detected in all cell lines. β-actin and topoisomerase IIβ were used as loading controls to discriminate the cytoplasmic and nuclear-enriched cell fractions, respectively. Representative of two independent experiments. **(c)** RD cells were transfected (Day 0) with EZH2 siRNA or control (CTR) siRNA and after 24 h transfected again (Day 1). Cells cultured in proliferating growth medium (GM, i.e. supplemented with 10% of fetal calf serum) were harvested and counted starting from 24 h from the first siRNA trasfection at the indicated time points. *P < 0.05 (Student’s t-test). Results from three independent experiments are shown; Bars, Standard Deviation (SD). **(d)** Western blot showing levels of EZH2 24 h and 48 h post-transfection with CTR or EZH2 siRNA in RD cells. β-actin served as loading control. Representative of four independent experiments. **(e)** Western blot showing histone H3 trimethylation on Lys27 (H3K27me3) and on Lys4 (H3K4me3) status 72 h after EZH2 or CTR siRNA transfection. Histone H3 was the loading control. Representative of three independent experiments.

We then evaluated the expression of EZH2 in 3 embryonal RMS cell lines. In agreement with results in primary samples, EZH2 expression is remarkably higher in these cell lines compared to control skeletal muscle precursors (SKMC), all cultured in a growth factor-enriched medium (supplemented with 10% serum) (Figure 2a). In particular, EZH2 appeared mostly localized in the nucleus (Figure 2b).

To define whether EZH2 was required to sustain embryonal RMS proliferation, as it occurs for other kind of human cancers [36,37], cell proliferation of the established embryonal RMS cell line RD, derived from a tumor recurrence [38], and cultured in growth medium, i.e. supplemented with 10% serum, was evaluated upon EZH2 genetic silencing. After two consecutive rounds of RNA interference with siRNAs against EZH2, the level of EZH2 protein expression in RD cells decreased more than 80% starting from 24 h after the first siRNA transfection (Figure 2d). In this condition, EZH2 knockdown in RD cells resulted in 36 ± 6% and 48 ± 8% inhibition of cell proliferation at day 3 and 4, respectively, compared to cells treated with a non-targeting control siRNA (Figure 2c). We confirmed the anti-proliferative effect of EZH2 siRNA with MTT assay (Additional file 2: Figure S1). To ascertain that the growth inhibition was the result of a reduced activity of EZH2, we analyzed the methylation status of Lys 27 on histone H3. Moreover, the Lys 4, a residue not methylated by EZH2, was also evaluated for methylation. We observed a global decrease of trimethylated Lys 27 (H3K27me3), but not of trimethylated Lys 4 (H3K4me3) at day 3 post-EZH2 siRNA transfection (Figure 2e), suggesting that EZH2-dependent histone methylation was specifically impaired upon EZH2 siRNA. These results indicate that over-expressed EZH2 sustains proliferation in embryonal RMS cells.

### Down-regulation of EZH2 is sufficient to restore embryonal RMS cell myogenic differentiation in growth medium

**Figure 3:**
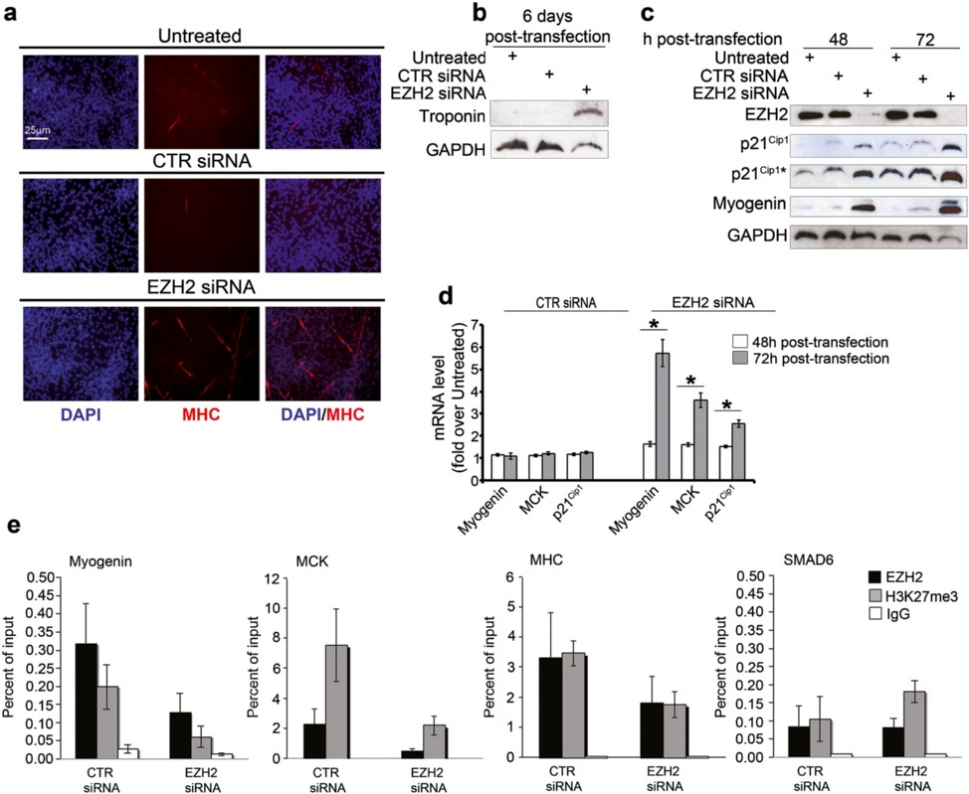
**Depletion of EZH2 results in myogenic differentiation of embryonal RD cells in growth medium (GM).** RD cells were transfected (t0) with EZH2 siRNA or control (CTR) siRNA and after 24 h silenced again. They were cultured in proliferating growth medium (GM, i.e. supplemented with 10% of fetal calf serum) for the following experimental procedures. **(a)** RD cells were analyzed for the induction of muscle-like differentiation 6 days post-siRNA transfection. Representative immunofluorescence showing de novo expression of endogenous Myosin Heavy Chain (MHC, red) in multinucleated fibers of EZH2 siRNA-transfected cells. DAPI was used for nuclear staining. Representative of four assays. **(b)** Western blot showing de novo expression of Troponin I 6 days post-siRNA transfection. GAPDH served as loading control. **(c)** Western blot showing EZH2, p21Cip1, Myogenin and GAPDH expression in RD cells 48 h and 72 h after EZH2 or CTR siRNA transfection and in untreated RD cells. (*band: longer exposure). Representative of four independent experiments. GAPDH served as loading control. **(d)** mRNA levels (real time qRT-PCR) of Myogenin, MCK, and p21Cip1 in RD cells 48 h and 72 h after EZH2 siRNA treatment were normalized to GAPDH levels and expressed as fold increase over untreated condition (1 arbitrary unit, not reported). Columns, means; Bars, SD. Results from three independent experiments are shown. *P < 0.05 (Student’s t-test). **(e)** ChIP assays on RD cells 72 h after EZH2 or CTR siRNA transfection showing the recruitment of EZH2 and the levels of histone H3 trimethylation on Lys27 (H3K27me3) on Myogenin, MCK, MHC and SMAD6 (as negative control) regulatory regions. Normal rabbit IgG were used as negative control. Graphs represent the percent of immunoprecipitated material relative to input DNA. Results are the average of three independent experiments. *P <0.05 (Student’s t-test).

Recent data showed that EZH2 down-regulation in RD cells induces partial recovery of myocyte phenotype after serum withdrawal [26]. Because of the inhibitory role of EZH2 in physiological myogenic differentiation, we asked whether the observed impaired proliferation of EZH2-depleted RD cells might be paralleled with the recovery of the myogenic fate even in the presence of 10% serum. We therefore set up differentiation assays on RD cells in the same culture condition of the proliferation assays, i.e. in growth medium, and analyzed the expression of differentiation markers. Six days after EZH2 siRNA transfection, multinucleated myotube-like structures positive for Myosin Heavy Chain (MHC) along with the expression of the skeletal muscle protein Troponin I, both indicative of terminal myogenic differentiation, were detected in EZH2-depleted RD cells compared to control siRNA cells (Figure 3a and 3b). Consistently, EZH2 knockdown induced the over-expression of both Myogenin and cyclin-dependent kinase inhibitor p21^Cip1^ (Figure 3c). Up-regulation of both Myogenin and the late differentiation marker Muscle Creatine Kinase (MCK) mRNA was detected as soon as 48 h post-EZH2 siRNA treatment, and was markedly enhanced after 72 h (Figure 3d). In line with the known inability of RD cells to undergo skeletal muscle-like differentiation under myogenic cues, the differentiation medium (low serum) culture condition was unable to potentiate the expression of Myogenin and the formation of MHC-positive multinucleated structures 72 h and 5 days post-siRNA transfection, respectively, as compared to growth (10% serum) medium condition (Additional file 3: Figure S2a and c). Similar results were obtained transfecting RD cells with a previously published siRNA that targets the 5′UTR of the endogenous EZH2 [31] (Additional file 3: Figure S2b and d), confirming EZH2 silencing-dependent effects.

**Figure 4:**
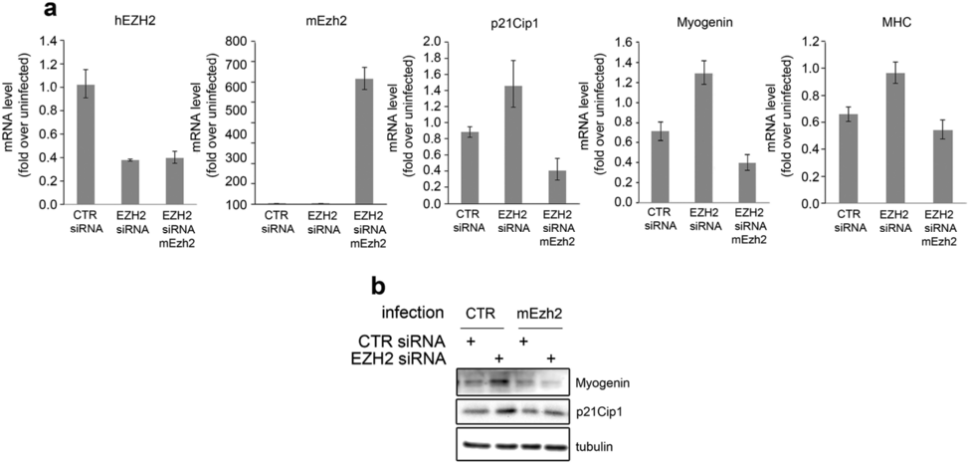
**Functional rescue of EZH2 depletion-dependent effects by overexpression of a murine Ezh2 in RD cells. (a)** mRNA levels (real time qRT-PCR) of p21Cip1, Myogenin and MHC in RD cells treated with CTR and EZH2 siRNA and then infected with a murine version of EZH2 (mEzh2) were normalized to GAPDH levels and expressed as fold increase over uninfected condition (1 arbitrary unit, not reported). mRNA levels of both human EZH2 (hEZH2) and murine EZH2 (mEzh2) are shown. Columns, means; Bars, SD. Results from three independent experiments are shown. *P < 0.05 (Student’s t-test). **(b)** Western blotting showing the rescuing effects of the overexpression of a murine EZH2 variant (mEzh2) on the levels of myogenin and p21Cip1 in RD cells previously treated with both CTR and EZH2 siRNA. α-tubulin was used as loading control.

In addition, RD cells were stably infected with a lentiviral vector expressing a short hairpin (sh)RNA against EZH2. Lentivirus-mediated EZH2 shRNA expression phenocopies the effects of EZH2 depletion by siRNA inducing the de-repression of p21^Cip1^, Myogenin and MCK genes, together with cell elongation and fusion to form multinucleated MHC-positive fibers compared to control shRNA (Additional file 4: Figure S3). To determine whether EZH2 directly represses muscle gene expression even in RD cells, as previously shown in myoblasts and RD cells in differentiation medium [22,23,26], we carried out ChIP assays to evaluate the binding of EZH2 and the Lys 27 histone H3 trimethylation status on muscle-specific loci. Figure 3e shows that EZH2 recruitment to regulatory regions of both early (i.e., *Myogenin*) and late (*MCK* and *MHC*) muscle-specific genes decreased in EZH2-silenced cells as compared to cells transfected with control siRNA. This correlated with a decrease in the levels of H3K27me3 at the indicated regulatory loci. Interestingly, the enrichment of EZH2 on late muscle genes (*MHC* and *MCK*) was 10-fold higher than those on the Myogenin locus under steady-state conditions (data not shown). This observation is consistent with the fact that RMS cells spontaneously express Myogenin, while they fail to produce MCK even when cultured in differentiation medium [8,9]. The functional effects of EZH2 knockdown on muscle genes and p21^Cip1^ expression were reverted by over-expression of a flag-tagged mouse Ezh2, indicating that they were specific for EZH2 (Figure 4). Altogether these results suggest that blocking EZH2 in actively growing embryonal RMS RD cells is a way to boost their cell-cycle exit to recover myogenic differentiation.

### Pharmacological inhibition of EZH2 prevents embryonal RMS cell proliferation

**Figure 5:**
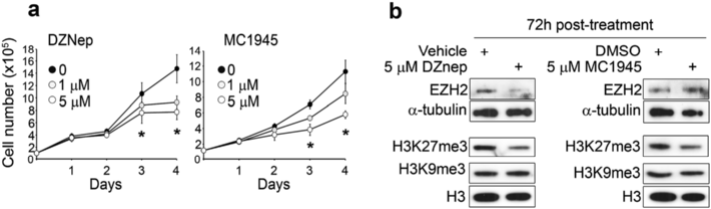
**Pharmacological inhibition of EZH2 prevents embryonal RMS cell proliferation. (a)** RD cells cultured in proliferating growth medium (GM, i.e. supplemented with 10% of fetal calf serum) were treated daily with either the S-adenosyl-L-homocysteine hydrolase inhibitor 3-deazaneplanocin A (DZNep) (left panels) or the EZH2 catalytic inhibitor MC1945 (right panels) at the reported concentrations or with vehicle (i.e., water for DZNep or DMSO for MC1945) and harvested and counted at the indicated time points. *P < 0.05 (Student’s t-test); Bars, SD. Three independent experiments in duplicate. **(b)** Western blot showing EZH2 along with histone H3 trimethylation on Lys27 (H3K27me3), and on Lys9 (H3K9me3) levels in RD cells treated for 72 h with 5 μM DZNep (left panel) and 5 μM MC1945 (right panel) or with vehicle (i.e., water or DMSO). Total H3 and - tubulin amounts were shown as the loading controls. Representative of three independent experiments.

To translate our results toward a future potential clinical intervention for aggressive embryonal RMS, we assessed the feasibility of pharmacological inhibition of EZH2 in RD cells. We treated RD cells with a well known EZH2 inhibitor, the S-adenosyl-L-homocysteine hydrolase inhibitor 3-Deazaneplanocin A (DZNep), which induces degradation of EZH2 [17,31,39]. In parallel, we used two new catalytic EZH2 inhibitors that inhibit the activity of the protein, the already validated EZH2 inhibitor MC1948 [28] and a new, more potent, derivative, MC1945 [32,40]. A significant reduction in the proliferation rate was noticed in RD cells treated for 72 h and 96 h with 1 μM of either DZNep or MC1945 compared to untreated or vehicle-treated cells (Figure 5a). Moreover, a significant greater inhibition of cell proliferation was obtained when RD cells were treated with 5 μM of each compound, suggesting a dose-dependent inhibitory effect (Figure 5a). These effects were accompanied by a down-regulation of EZH2 protein levels upon DZNep treatment (Figure 5b, left panel) whereas the levels remained constant after treatment with the catalytic inhibitors MC1945, as expected (Figure 5b, right panel) [28]. Both DZNep and MC1945 treatments resulted in a decrease in global levels of the EZH2 repressive mark H3K27me3 (Figure 5b) (28–30). On the contrary, the levels of H3K9me3, another repressive mark, remained unchanged after both treatments, demonstrating the specificity of the two compounds in targeting EZH2-containing complexes in our experimental conditions (Figure 5b). Same results were obtained in preliminary experiments with MC1948 (Additional file 5: Figure S4a and b). Similarly to what happened for EZH2-silenced cells, culture condition in differentiation medium (low serum) was unable to significantly potentiate the formation of MHC-positive multinucleated structures 4 days post-treatment as compared to growth (10% serum) medium condition (Additional file 6: Figure S5). By contrast, 5 days of treatment in DM lead to detachment of cells from the well surface, maybe due to cytotoxic effects of nutrient-deprived conditions (data not shown).

Altogether, these findings clearly suggest that pharmacological inhibition of EZH2 affects the proliferative potential of embryonal RMS cells and phenocopies the cell-specific effect of siRNA-mediated EZH2 depletion.

### Pharmacological inhibition of EZH2 restores myogenic differentiation of embryonal RMS cells even in the presence of growth medium

**Figure 6:**
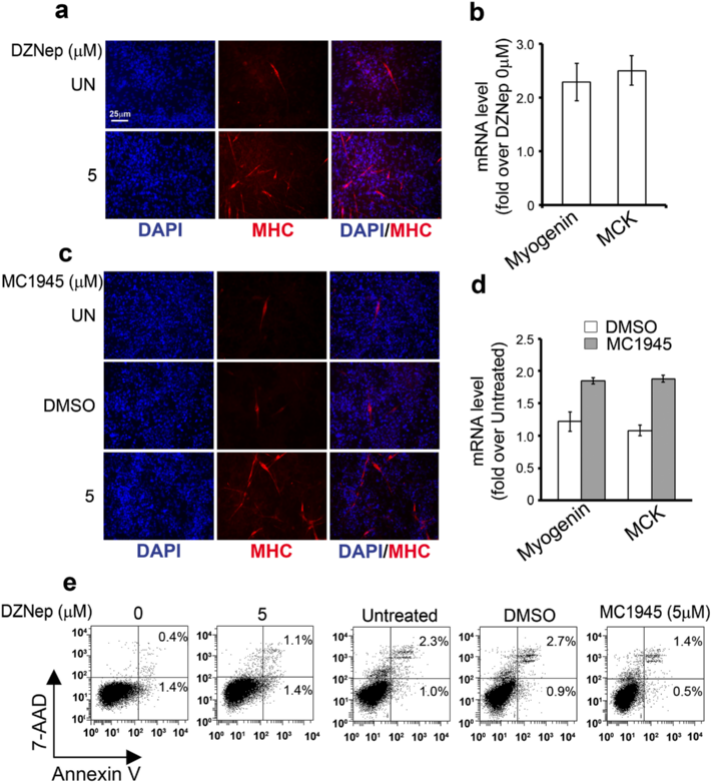
**Pharmacological inhibition of EZH2 restores myogenic differentiation of embryonal RMS cells in the presence of growth medium (GM).** RD cells were analyzed for the induction of muscle-like differentiation after 6 days of 5 μM DZNep **(a)** and MC1945 **(c)** treatments. Representative immunofluorescence showing de novo expression of endogenous Myosin Heavy Chain (MHC, red) in multinucleated fibers of DZNep and MC1945 treated RD cells. Untreated (UN) and control cells treated with vehicle (i.e., water or DMSO) are shown. Representative immunofluorescence of three assays. mRNA levels (real time qRT-PCR) of Myogenin and MCK in RD treated for 72 h with 5 μM DZNep **(b)** and 5 μM MC1954 **(d)** were normalized to GAPDH levels and expressed as fold increase over Untreated condition (1 arbitrary unit, not reported). Columns, means; Bars, SD. Results from two independent experiments are shown. **(e)** RD cells Untreated or treated for 96 h with DZNep (left) or MC1945 (right) at the indicated concentrations were stained for Annexin V and 7-AAD, and the frequency of Annexin V and 7-AAD-positive labeling (% cell death) was recorded by flow cytometry. Representative cytofluorometric plots are shown. Annexin V+/7-AAD- events (lower right quadrants) represent early stages of apoptosis, whereas Annexin V+/7-AAD + events (upper right quadrants) stand for late apoptotic cells. Representative of three independent experiments run in duplicate.

In order to evaluate whether the strong inhibitory effects on RD proliferation obtained by blocking EZH2 methyltransferase activity was associated to the triggering of myogenic-like differentiation we treated RD cells with 1 μM of MC1948 for 6 days and then we analyzed myogenic differentiation by immunocytochemistry. We noticed the appearance of multinucleated myotube-like structures expressing MHC in RD cells treated with MC1948 compared to vehicle-treated cells (Additional file 4: Figure S3c). Then we extended the study enrolling DZNep and MC1945. Treatment of RD cells for 6 days with either 5 μM of DZNep or MC1945 resulted in the formation of MHC-positive multinucleated myotube-like structures (Figure 6a and 6c) and in the induction of *Myogenin* and *MCK* gene transcription 72 h post-treatment (Figure 6b and 6d). Consistently with these results, no sign of apoptosis testified by the lack of appearance of apoptotic Annexin V-positive cells was evidenced in both DZNep- and MC1945-treated RD cells (Figure 6e). Altogether, these results suggest that the two pharmacological inhibitory approaches of EZH2 function are capable to restore myogenic differentiation of embryonal RMS cells as occurs in the case of EZH2 genetic depletion.

### Pharmacological inhibition of EZH2 induces myogenic differentiation in embryonal RMS tumor xenografts

**Figure 7:**
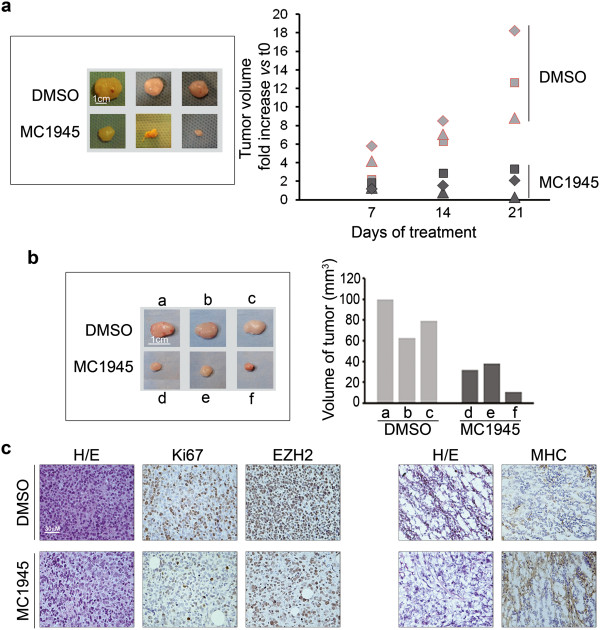
**Analysis of pharmacological inhibition of EZH2 *in vivo*.** RD cell suspensions in PBS (10×10^6^ cells) were injected subcutaneously into the posterior flanks of athymic 6-week-old female BALB/c nude mice (nu + \nu+). **(a)** As soon as the tumors became palpable, i.e., about 2 months after the initial inoculation when they reached approximately 70–80 mm^3^, mice were intraperitoneally injected with MC1945 (2.5 mg/Kg) or control vehicle (DMSO) twice daily, 3 days per week for 3 weeks when mice were sacrificed with no visible signs of toxicity. Tumor volume was measured by caliper weekly for 21 days (right) after which xenografts were surgically removed (left). **(b)** for immunohistochemical studies, a group of mice were sacrificed at day 12 of treatment, during the exponential tumor growth phase, and xenografts excised. Tumor volume was measured by caliper prior to the initiation of treatment and at the time of sacrifice. (**b**, left) xenografts from either nude mice treated with MC1945 (d, e, f) or with DMSO (a, b, c) as controls. (**b**, right) Portions of the excised tumors in **(b)** were embedded in paraffin for immunohistochemical analysis or snap-frozen in liquid N_2_. Histogram reports tumor volumes of each xenograft. **(c)** Sections of 10 μm cut from xenograft blocks were stained with hematoxylin/eosin. Five μm serial sections were subjected to immunohistochemistry for the expression of EZH2 and Ki67 (paraffin-embedded, nuclear orange staining in left panels) and MHC (OCT embedded, staining with the MF-20 antibody, cytoplasmic orange staining in right panels) of serial DMSO (upper panels) and MC1945 treated (lower panels) sections from RD xenografts. Counterstaining was carried out with Gill’s hematoxyline (Bio-Optica, MI, Italy). Sections were dehydrated and mounted in mounting medium. Images were acquired under an Eclipse E600 microscope (Nikon) through the LUCIA software, version 4.81 (Nikon) with a Nikon Digital Camera DXM1200F.

To verify that inhibiting EZH2 with the catalytic inhibitor MC1945 might reduce RMS cell proliferation and concomitantly induce myogenic differentiation even *in vivo*, we injected nude mice subcutaneously with RD cells and, when tumors began palpable, intraperitoneally injected them with 2.5 mg/kg of MC1945 or with vehicle (DMSO). MC1945 treatment resulted in a significant reduction in xenograft tumor growth after 3 weeks (Figure 7a). The myogenic differentiation was analyzed in xenografts excised 12 days after the beginning of the treatment, during the exponential growth phase (Figure 7b and c). The effects of the EZH2 inhibitor were anti-proliferative, as demonstrated by the retardation of tumor growth (Figure 7a and b) associated to a reduction of the proliferative marker Ki67 in tumor xenografts (Figure 7c, left panel), and led to de novo expression of fibers positive for Myosin Heavy Chain (MHC) compared to vehicle treatment (Figure 7c, right panel). These findings provide evidence that it is possible to pharmacologically counteract the tumorigenic function of EZH2 *in vivo*, and that the treatment could promote a more differentiated phenotype directly into the tumor bulk.

## Discussion

In the last decade, to trace the way for developing innovative anti-cancer therapies, several groups focused their pre-clinical research on the modulation of epigenetic regulators often aberrantly expressed in cancer. Due to the fact that epigenetic processes are key players in cell tissue specification during the embryonal life, this approach seems to be particularly captivating for those cancers, such as pediatric embryonal RMS, in which the pathogenic mechanisms involve the deregulation of genes controlling the lineage commitment [41]. Among these, the histone methyltransferase EZH2 is a fundamental negative regulator of myogenic precursor differentiation by repressing the expression of myogenic genes through the H3K27me3 mark deposition on the promoters of myogenic genes [22,28]. We recently reported that EZH2 transcripts were aberrantly expressed in both embryonal RMS primary tumors and in the RD cell line [25,35]. In this study, we report that, as for transcripts, EZH2 protein is aberrantly over-expressed in 19 out of 19 embryonal RMS primary tumors compared to normal muscle tissues, thus indicating that the high level of expression of EZH2 is a common molecular lesion of embryonal RMS neoplasia.

Moreover, a recent report indicates that the RD cell line, derived from an embryonal RMS local recurrence and thus representative of an aggressive tumor [38], may reactivate muscle-specific genes and develop a partial recovery of myocyte phenotype following EZH2 knockdown when depleted of serum [26]. We show here that it is possible to revert the tumor phenotype of the RD cell line by silencing EZH2 even under proliferative stimuli such as in a serum-enriched molecular context. The final result is the acquisition of a myogenic phenotype, by the de-repression of myogenic genes *Myogenin* and *MCK*, which can be rescued by the over-expression of a murine Ezh2 not targeted by the used siRNA oligos. More importantly, as a proof-of-concept we report that in these pro-proliferative conditions, pharmacological inhibition of EZH2 by two different approaches, *i.e.* by decreasing its availability or hampering its activity, is capable to prevent the proliferation and allow the recovery of myogenic differentiation of these cells *in vitro* and *in vivo*. In line with the inability of RD cells to undergo terminal differentiation in conditions that induce myotube formation in normal, non-tumorigenic, myoblasts (REF), low-serum differentiation medium did not potentiate the effect of EZH2 depletion/inactivation on the myogenic-like characteristics *vs* growth medium. Consistently, EZH2 expression is not modulated by serum deprivation in RD cells (data not shown). Small molecule inhibitors of histone methyltransferases are emerging [42] and a number of novel EZH2 inhibitors are under preclinical evaluation in other types of cancer [43-45].

Here we treated RD RMS cells with the prototype inhibitor of PRC2, deazaneplanocin A (DZNep), which acts through an indirect mechanism by reducing the level of EZH2 protein [17,46]. Recently, DZNep has been reported to be effective in several preclinical studies favoring apoptosis and/or differentiation of tumor cells [39,47-49]. We found that DZNep arrested RD proliferation in a dose-dependent manner with a concomitant down-regulation of EZH2 protein levels and a decrease in global levels of H3K27me3, while the levels of the other repressive mark H3K9me3 remained unchanged, suggesting an EZH2-specific effect at the doses utilized. Strikingly, in the same growth condition DZNep induced the appearance of MHC-positive multinucleated myotube-like structure in RD cells, accompanied by the activation of myogenic genes such as *Myogenin* and *MCK*, and with no signs of apoptosis. The observation that in RMS DZNep induces myogenic differentiation instead of apoptosis, the general effect that DZNep has in other human cancer, suggests that its inhibition toward EZH2 is quite specific being pro-differentiative. However, since DZNep may affect other methyltransferases, we enrolled in our study also two molecules belonging to a new class of catalytic inhibitors, validated against a panel of histone methyltransferases [32,40], MC1948, which has been already validate as EZH2 inhibitor in myoblasts [28] and a new, more effective, derivative, MC1945. Both MC inhibitors phenocopied the effects of DZNep and EZH2 genetic depletion *in vitro*, indicating a common mechanism of action. More importantly we observed that MC1945 was able to restrain tumor growth of RD xenografts in nude mice inducing tumor cells differentiation *in vivo*. Pharmacological inhibition of EZH2 by using a new EZH2 inhibitor has been recently shown to induce anti-tumoral effects in malignant rhabdoid tumor (MRT) cells deleted for *SMARCB1*[50]. Importantly, this result highlights the dependency of *SMARCB1*-mutant/deleted MRT tumorigenicity on EZH2. However, the Authors showed no effects of the inhibitor on *SMARCB1*-wild-type RD cells that were cultured in medium replenished with the drug on day 4 [50]. Differently, we treated RD cells with new doses of inhibitors every day since this approach was defined as effective during preliminary experiments. As a consequence, in our experimental protocol tumor cells were in contact with fresh drug each 24 h. These diverse approaches could be responsible for the difference in the response to pharmacological inhibitors.

In summary, here we present a preclinical study in which the experimental evidence indicates that the pharmacological targeting of EZH2 might represent a way to reduce the aggressiveness of RMS, promoting a more differentiated phenotype and thus enlarging the scenery of the future clinical intervention to treat this type of tumors.

## Conclusions

Collectively our data provide evidence that EZH2 abnormal over-expression is responsible for both sustaining proliferation and inhibiting myogenic differentiation of embryonal RMS. More importantly, our results indicate that pharmacological targeting of EZH2 might represent a potential feasible approach to be used as adjuvant treatment for making conventional therapy more effective on less aggressive and more differentiated RMS.

## Abbreviations

RMS: Rhabdomyosarcoma; SKMC: Skeletal Muscle Cells; GM: Growth factor-supplemented Medium; EZH2: Enhancer of Zeste of Homologue 2; STS: Soft-Tissue-Sarcoma; PcG protein: Polycomb Group protein; PRC2: Polycomb Repressor Complex 2; siRNA: small interfering RNA; (sh)RNA: short-hairpin RNA; MHC: Myosin Heavy Chain; MCK: Muscle Creatine Kinase; Ado-Hcy: S-adenosylhomocysteine; DZNep: S-adenosylhomocysteine hydrolase inhibitor 3-deazaneplanocin A; SAM: S-adenosylmethionine; MC1948 and MC1945: catalytic EZH2 inhibitors; H3K27me3: trimethylated lysine 27 on Histone 3; H3K4me3: trimethylated lysine 4 on Histone 3; H3K9me3: trimethylated lysine 9 on Histone 3.

## Competing interests

The authors indicate no competing financial interests.

## Authors’ contributions

RC participated in the design of the study, participated in statistical analysis and in manuscript writing. RC, EC, LA, GB, PPL, AD and FV participated in the *in vitro* experiments. MDS participated in the *in vitro* and *in vivo* studies. GMM, RB and AI carried out primary samples and clinical data collection of RMS patients. SS and IS participated with reagents and discussion. VEM, SV and AM produced DZNep and MC inhibitors and participated with discussion and data analysis. PLP was involved in the design of the study, wrote and reviewed the manuscript. FL participated in the design of the study, discussion of clinical and research data and reviewed the manuscript. DP participated in the study design, experimental procedures, data analysis and manuscript writing. RR was the responsible of the conception and design of the study, coordinated the study, was involved in manuscript writing and reviewed the final version. LA, MDS and GB contributed equally as second co-authors. All authors read and approved the manuscript.

## Authors’ informations

RC is a Junior Scientist and LA, GB, MDS, PPL and FV are PhD and doctoral fellows working on the transcriptional regulation of pediatric cancers in the Laboratory of Angiogenesis directed by RR. DP is a Junior Scientist and EC is a doctoral fellow working on the role of developmental pathway in rhabdomyosarcoma. AD is a doctoral fellow working on the developmental mechanisms in muscle cells in the Laboratory directed by PLP. GMM, RB and AI are Oncologist, Pathologist and Surgeon of the oncology group, respectively. SS is a PhD and Full Professor in Neuroscience and a Chercheur National; Fonds de la Recherche en Sante du Quebec. IS is a MD and Full Professor committed to the preclinical and clinical research against pediatric cancers. VEM and AM are MD and Full Professors in Biochemistry with long lasting experience in biochemical drugs production and testing. SV is a PhD working in the Laboratory directed by AM. PLP is a MD and Full Professor with long lasting experience in the study of skeletal muscle and soft tissue sarcomas. FL is an MD and Full Professor of Pediatrics and the Head of the Oncohematology Dept with a long standing experience in preclinical research and clinical management of pediatric tumor patients. RR is a PhD and the Head of the Laboratory of Angiogenesis with experience in mechanisms that regulate gene expression and cell growth in pediatric cancers.

## Pre-publication history

The pre-publication history for this paper can be accessed here:

http://www.biomedcentral.com/1471-2407/14/139/prepub

## Supplementary Material

Additional file 1 Supplementary Materials and Methods. Click here for file

Additional file 2: Figure S1 Biochemical analysis of cell viability using MTT assay shows the anti-proliferative effect of EZH2 siRNA in RD cells. RD cells treated with either CTR siRNA or EZH2 siRNA were cultured in proliferating growth medium (GM, *i.e.*, supplemented with 10% fetal calf serum) for 3 days and then incubated with MTT (3-(4,5-Dimethylthiazol-2-yl)-2,5-diphenyltetrazolium bromide, a tetrazole) reagent (Sigma Chemical Co., St Louis, MO, USA), according to manufacturer’s recommendations. *P<0.05 (Student’s t-test); Columns, means; Bars, SD. Results from three independent experiments are shown. Click here for file

Additional file 3: Figure S2 Differentiation medium (DM, *i.e.*, supplemented with 2% horse serum) does not potentiate EZH2 depletion-dependent myogenic-like differentiation of embryonal RD cells compared to proliferating growth medium (GM, *i.e.*, supplemented with 10% fetal calf serum). RD cells were transfected (t0) either with EZH2 siRNA or control (CTR) siRNA or with an EZH2 siRNA targeting the 5′UTR of the endogenous EZH2 (EZH2 siRNA*) or the corresponding control siRNA (CTR siRNA*) and after 24 h silenced again. Cells were cultured in either GM or DM and media were replenished every two days. (a) Western blot showing EZH2 and Myogenin in RD cells 72 h after EZH2 siRNA or CTR siRNA transfection and in untreated RD cells. Representative of two independent experiments. GAPDH served as loading control. (b, left panels) Western blot showing EZH2 and H3K27me3 levels in RD cells silenced with EZH2 siRNA* or CTR siRNA* 72 h after siRNA transfection. (b, right panels) Western blot showing EZH2 and Myogenin in RD cells 72 h after EZH2 siRNA* or CTR siRNA* transfection and in untreated cells. Representative of two independent experiments. GAPDH served as loading control. (c, d) RD cells were silenced for EZH2 and analyzed for the induction of muscle-like differentiation 5 days post-siRNA transfection. Media were replenished every 2 days. Representative immunofluorescence showing *de novo* expression of endogenous Myosin Heavy Chain (MHC, green; Alexa Fluor 488 secondary antibody, #A11017: Invitrogen, Carlsbad, CA) in multinucleated fibers of EZH2-depleted cells using (c) EZH2 siRNA and (d) an EZH2 siRNA targeting the 5′UTR of the endogenous EZH2 (EZH2 siRNA*) and their corresponding control siRNAs . DAPI (blue) was used for nuclear staining. Representative of two assays. Click here for file

Additional file 4: Figure S3 Effects of EZH2 depletion by short-hairpin (shRNA) expression in RD cells cultured in proliferating growth medium (GM, i.e., supplemented with 10% fetal calf serum). RD cells were infected with lentiviral vectors expressing either a shRNA against EZH2 (EZH2 shRNA) or a non-targeting control shRNA (CTR shRNA). (a, left) mRNA levels (real time qRT-PCR) of EZH2, Myogenin and MCK in EZH2 shRNA RD cells both 4 days post-infection and after 30 days of selection in puromycin (stably shRNA-expressing cells). Values were normalized to GAPDH levels and expressed as fold increase over CTR shRNA (1 arbitrary unit, not reported). Columns, means; Bars, SD. Results from two independent experiments are shown. (a, right) Western blot showing levels of EZH2 in stably expressing EZH2 and CTR shRNA RD cells. α-tubulin served as loading control. Representative of two independent experiments. (b) Stably EZH2 and CTR shRNA-expressing RD cells were analyzed for their myogeinc potential. Representative immunofluorescence showing de novo expression of endogenous Myosin Heavy Chain (MHC, red) in multinucleated fibers of EZH2 shRNA-expressing cells after 6 days in culture. Representative of two assays. Click here for file

Additional file 5: Figure S4 Inhibiting EZH2 in RD cells by using the catalytic inhibitor MC1948. (a) RD cells were treated with 5 μM MC1948 or DMSO (vehicle) in proliferating growth medium (GM, i.e. supplemented with 10% of fetal calf serum), harvested and counted at the indicated time points starting at 24 h (day 1) from the onset of treatment. *P<0.05 (Student’s t-test); Bars, Standard Deviation (SD). (b) Western blot showing levels of histone H3 trimethylation on Lys27 (H3K27me3) and EZH2 after 5 μM MC1948 treatment RD cells. Total Histone3 (H3) and α-tubulin served as loading controls. Representative of 3 independent experiments. (c) RD cells were analyzed for the induction of muscle-like differentiation by immunofluorescence for Myosin Heavy Chain (MHC) protein after 6 days of 5 μM MC1948 treatment. Control cells treated with DMSO (vehicle) are shown. Representative immunofluorescence of three assays. Click here for file

Additional file 6: Figure S5 Differentiation medium (DM, *i.e.*, supplemented with 2% horse serum) does not potentiate myogenic-like differentiation of embryonal RD cells upon pharmacologic inhibition of EZH2 compared to proliferating growth medium (GM, *i.e.*, supplemented with 10% fetal calf serum). Representative phase-contrast images of RD cells analyzed for the induction of skeletal muscle-like phenotype after 4 days of either DZNep or MC1945 (5 μM both) treatment in GM or DM. White arrows indicate multinucleated fibers. Control cells treated with vehicle (i.e., water (UN) or DMSO) are shown. Magnification 200×. Representative of two assays. Click here for file
