# Supplementary material for: Pharmacological inhibition of EZH2 as a promising differentiation therapy in embryonal RMS
Source: BMC Cancer. 2014 Feb 27;14:139. doi: 10.1186/1471-2407-14-139 (PMC4016511; doi:10.1186/1471-2407-14-139)
Supplement: Additional file 1 — Supplementary Materials and Methods. [file 1471-2407-14-139-S1.doc]

**Supplementary Materials and Methods:**

**Differentiation medium cell culture**

To mimic differentiation conditions and compare experimental results showed in growth medium, RD (ERMS) cells were cultured in Dulbecco Modified Eagle’s Medium (Euroclone, Pero, MI, Italy) supplemented with 2% horse serum, 1% glutamine and 1% penicillin-streptomycin (DM, differentiation medium).

**Transient EZH2 silencing with an additional oligo siRNA**

Cells were sequentially transfected by 2 subsequent rounds (24h) with a siRNA targeting the 5’-UTR of EZH2 mRNA (Wu et al. Ref 30) with the following sequence 5’-CGGTGGGACTCAGAAGGCA-3’ (100 nM final concentration each round) (Sigma-Proligo, St Louis, MO) using Oligofectamine (Invitrogen, Carlsbad, CA), according to manufacturer’s recommendations. A non-targeting siRNA was used as control (Sigma, St Louis,MO).

**Prolonged RNA interference with shRNA**

Short hairpin RNA (sh)RNA-mediated silencing was performed by infecting RD cells with three different Smart Choice Lentiviral shRNA particles expressing 19 nucleotide shRNAs against EZH2 (VSH5417, SH-004218-01-01, EZH2 target sequence TTTGCAAATCATTCCGGTAA) (Dharmacon, Thermo Fisher Scientific, Lafayette, CO). A non-targeting shRNA (SV 2.0; HV221111) was used as negative control (Dharmacon, Thermo Fisher Scientific, Lafayette, CO). Cells were infected with 5 multiplicity of infection (MOI) particles per cell of each lentivirus using polybrene (Invitrogen, Carlsbad, CA) at final concentration of 0,6 µg /ml for 16 hours in medium supplemented with 10% FCS. EZH2 silencing in RD cells was assessed by both Western blotting and qRT-PCR after 72h post-transfection. Stable shRNAs RD cells were obtained after 4 weeks of selection with puromycin (2.5µg/ml).
