# Supplementary figures and images for: Pharmacological inhibition of EZH2 as a promising differentiation therapy in embryonal RMS
Source: BMC Cancer. 2014 Feb 27;14:139. doi: 10.1186/1471-2407-14-139 (PMC4016511; doi:10.1186/1471-2407-14-139)

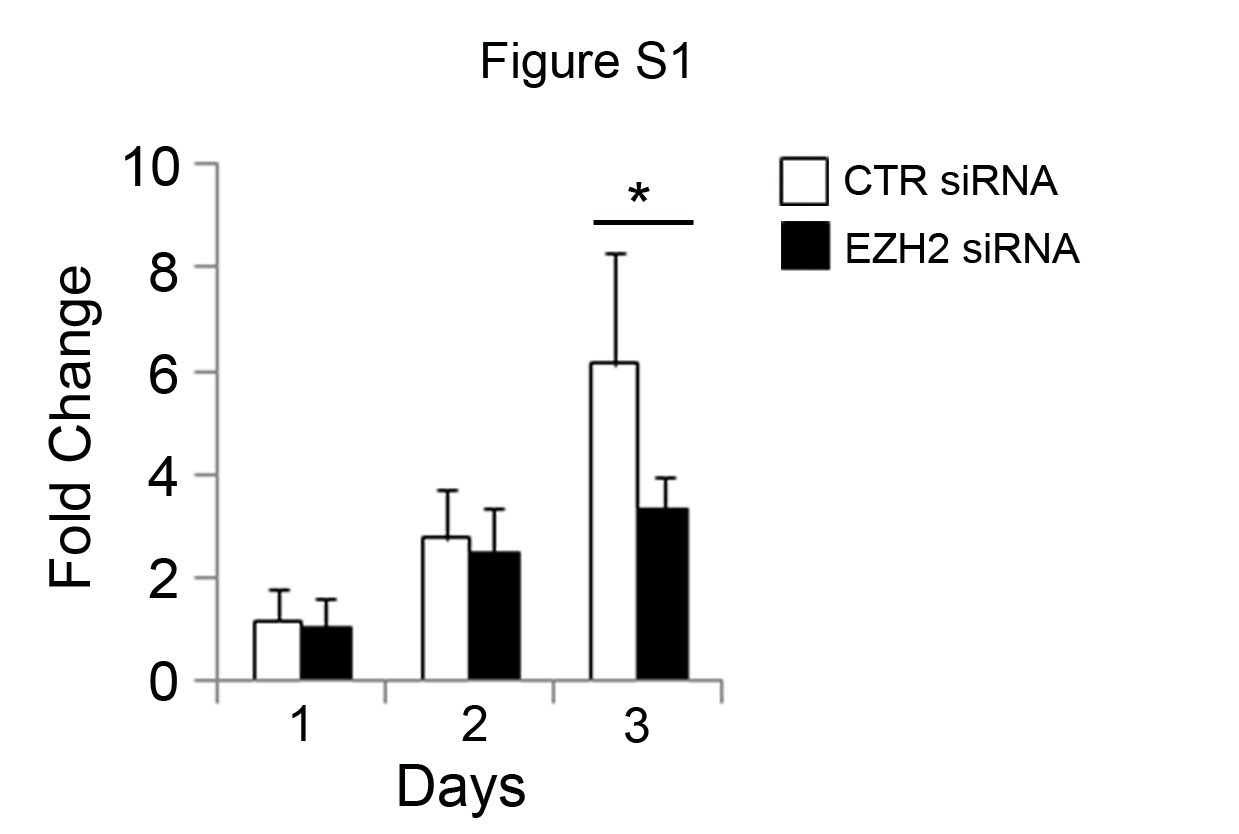

Supplement: Additional file 2: Figure S1 — Biochemical analysis of cell viability using MTT assay shows the anti-proliferative effect of EZH2 siRNA in RD cells. RD cells treated with either CTR siRNA or EZH2 siRNA were cultured in proliferating growth medium (GM, i.e., supplemented with 10% fetal calf serum) for 3 days and then incubated with MTT (3-(4,5-Dimethylthiazol-2-yl)-2,5-diphenyltetrazolium bromide, a tetrazole) reagent (Sigma Chemical Co., St Louis, MO, USA), according to manufacturer’s recommendations. *P<0.05 (Student’s t-test); Columns, means; Bars, SD. Results from three independent experiments are shown. [file 1471-2407-14-139-S2.jpeg]

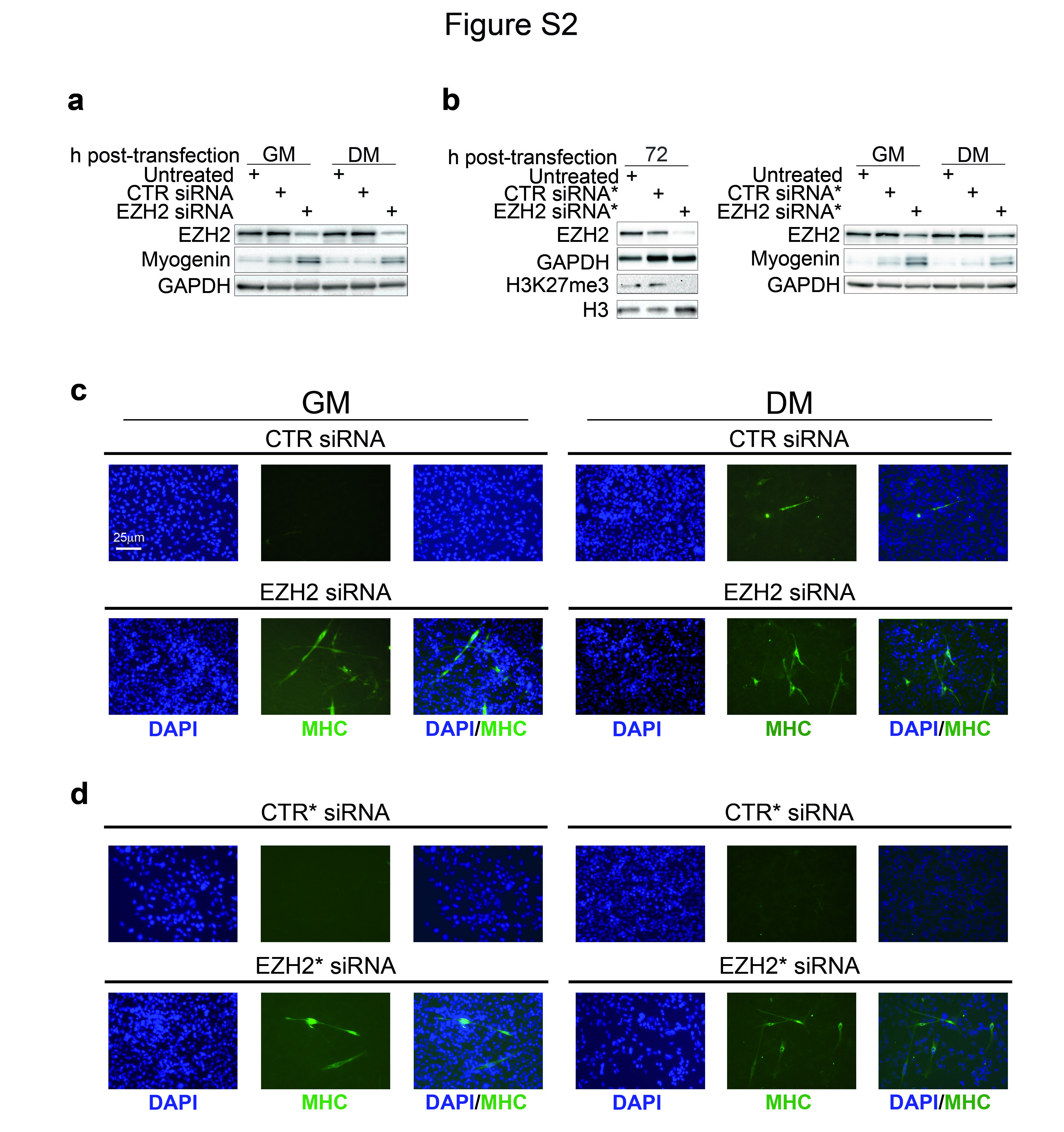

Supplement: Additional file 3: Figure S2 — Differentiation medium (DM, i.e., supplemented with 2% horse serum) does not potentiate EZH2 depletion-dependent myogenic-like differentiation of embryonal RD cells compared to proliferating growth medium (GM, i.e., supplemented with 10% fetal calf serum). RD cells were transfected (t0) either with EZH2 siRNA or control (CTR) siRNA or with an EZH2 siRNA targeting the 5′UTR of the endogenous EZH2 (EZH2 siRNA*) or the corresponding control siRNA (CTR siRNA*) and after 24 h silenced again. Cells were cultured in either GM or DM and media were replenished every two days. (a) Western blot showing EZH2 and Myogenin in RD cells 72 h after EZH2 siRNA or CTR siRNA transfection and in untreated RD cells. Representative of two independent experiments. GAPDH served as loading control. (b, left panels) Western blot showing EZH2 and H3K27me3 levels in RD cells silenced with EZH2 siRNA* or CTR siRNA* 72 h after siRNA transfection. (b, right panels) Western blot showing EZH2 and Myogenin in RD cells 72 h after EZH2 siRNA* or CTR siRNA* transfection and in untreated cells. Representative of two independent experiments. GAPDH served as loading control. (c, d) RD cells were silenced for EZH2 and analyzed for the induction of muscle-like differentiation 5 days post-siRNA transfection. Media were replenished every 2 days. Representative immunofluorescence showing de novo expression of endogenous Myosin Heavy Chain (MHC, green; Alexa Fluor 488 secondary antibody, #A11017: Invitrogen, Carlsbad, CA) in multinucleated fibers of EZH2-depleted cells using (c) EZH2 siRNA and (d) an EZH2 siRNA targeting the 5′UTR of the endogenous EZH2 (EZH2 siRNA*) and their corresponding control siRNAs . DAPI (blue) was used for nuclear staining. Representative of two assays. [file 1471-2407-14-139-S3.jpeg]

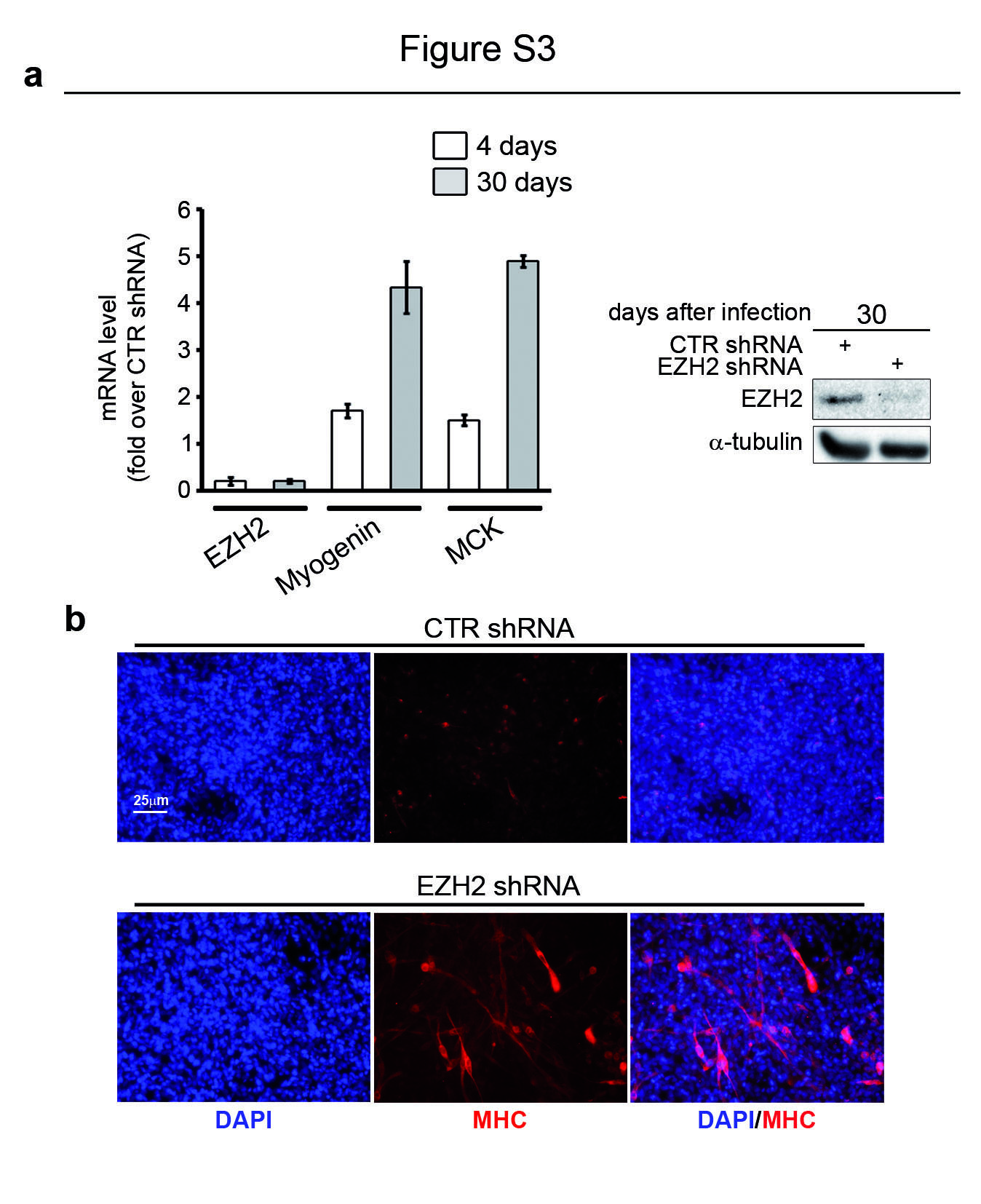

Supplement: Additional file 4: Figure S3 — Effects of EZH2 depletion by short-hairpin (shRNA) expression in RD cells cultured in proliferating growth medium (GM, i.e., supplemented with 10% fetal calf serum). RD cells were infected with lentiviral vectors expressing either a shRNA against EZH2 (EZH2 shRNA) or a non-targeting control shRNA (CTR shRNA). (a, left) mRNA levels (real time qRT-PCR) of EZH2, Myogenin and MCK in EZH2 shRNA RD cells both 4 days post-infection and after 30 days of selection in puromycin (stably shRNA-expressing cells). Values were normalized to GAPDH levels and expressed as fold increase over CTR shRNA (1 arbitrary unit, not reported). Columns, means; Bars, SD. Results from two independent experiments are shown. (a, right) Western blot showing levels of EZH2 in stably expressing EZH2 and CTR shRNA RD cells. α-tubulin served as loading control. Representative of two independent experiments. (b) Stably EZH2 and CTR shRNA-expressing RD cells were analyzed for their myogeinc potential. Representative immunofluorescence showing de novo expression of endogenous Myosin Heavy Chain (MHC, red) in multinucleated fibers of EZH2 shRNA-expressing cells after 6 days in culture. Representative of two assays. [file 1471-2407-14-139-S4.jpeg]

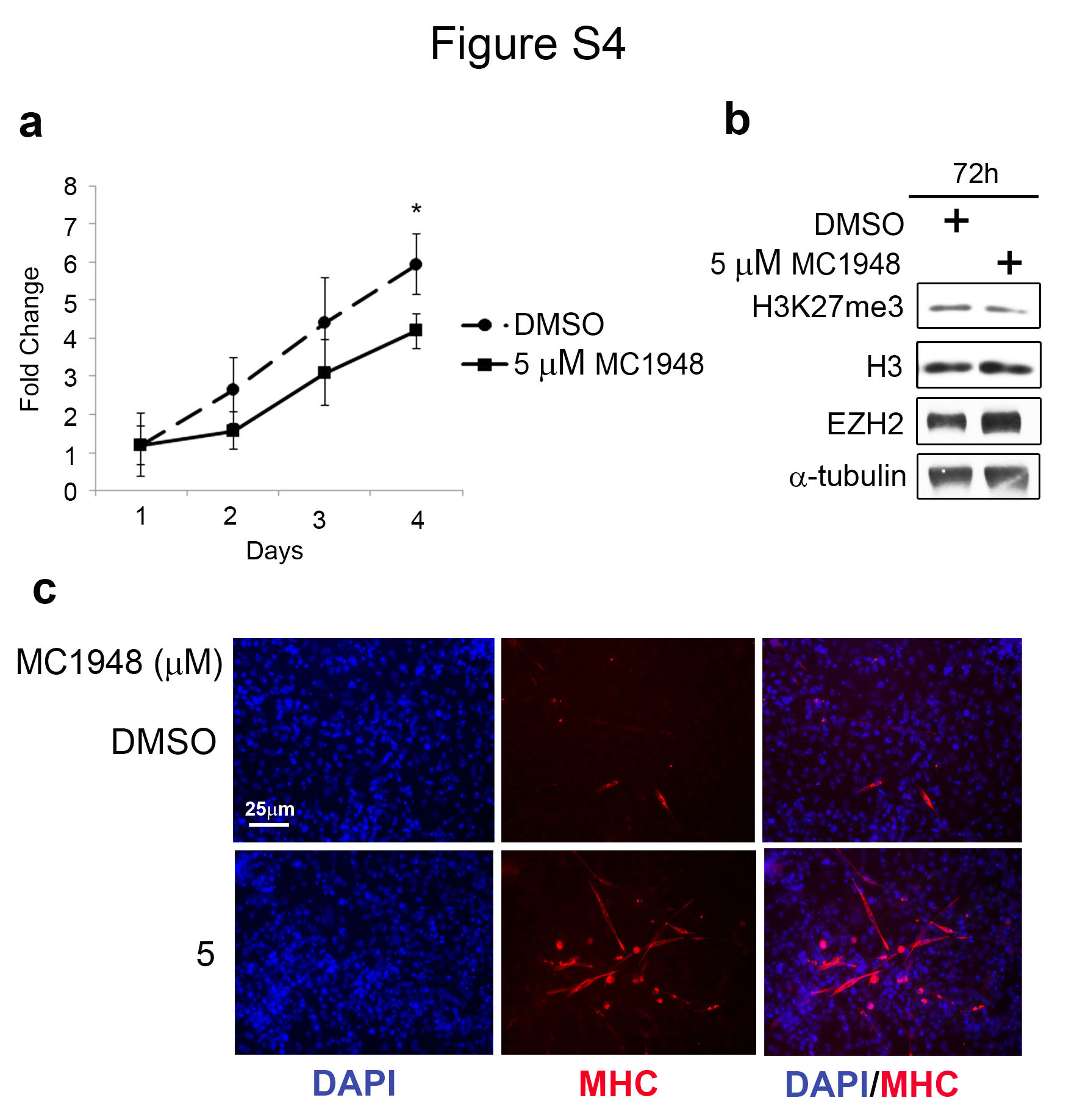

Supplement: Additional file 5: Figure S4 — Inhibiting EZH2 in RD cells by using the catalytic inhibitor MC1948. (a) RD cells were treated with 5 μM MC1948 or DMSO (vehicle) in proliferating growth medium (GM, i.e. supplemented with 10% of fetal calf serum), harvested and counted at the indicated time points starting at 24 h (day 1) from the onset of treatment. *P<0.05 (Student’s t-test); Bars, Standard Deviation (SD). (b) Western blot showing levels of histone H3 trimethylation on Lys27 (H3K27me3) and EZH2 after 5 μM MC1948 treatment RD cells. Total Histone3 (H3) and α-tubulin served as loading controls. Representative of 3 independent experiments. (c) RD cells were analyzed for the induction of muscle-like differentiation by immunofluorescence for Myosin Heavy Chain (MHC) protein after 6 days of 5 μM MC1948 treatment. Control cells treated with DMSO (vehicle) are shown. Representative immunofluorescence of three assays. [file 1471-2407-14-139-S5.jpeg]

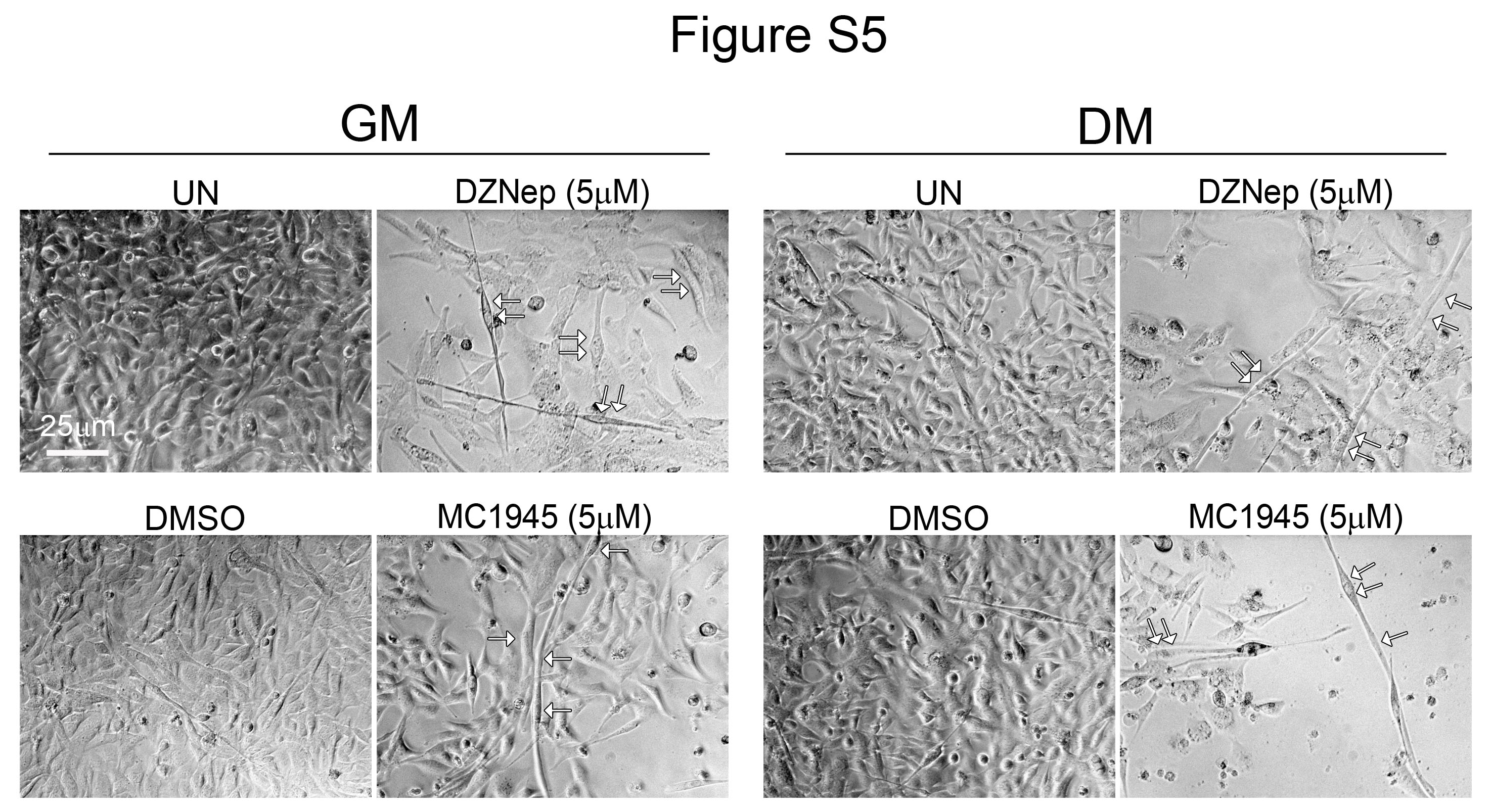

Supplement: Additional file 6: Figure S5 — Differentiation medium (DM, i.e., supplemented with 2% horse serum) does not potentiate myogenic-like differentiation of embryonal RD cells upon pharmacologic inhibition of EZH2 compared to proliferating growth medium (GM, i.e., supplemented with 10% fetal calf serum). Representative phase-contrast images of RD cells analyzed for the induction of skeletal muscle-like phenotype after 4 days of either DZNep or MC1945 (5 μM both) treatment in GM or DM. White arrows indicate multinucleated fibers. Control cells treated with vehicle (i.e., water (UN) or DMSO) are shown. Magnification 200×. Representative of two assays. [file 1471-2407-14-139-S6.jpeg]
